# Pediatric precursor B acute lymphoblastic leukemia: are T helper cells the missing link in the infectious etiology theory?

**DOI:** 10.1186/s40348-017-0072-z

**Published:** 2017-05-16

**Authors:** Simone Bürgler, David Nadal

**Affiliations:** Experimental Infectious Diseases and Cancer Research, University Children’s Hospital Zürich, 8008 Zürich, Switzerland

**Keywords:** Precursor B acute lymphoblastic leukemia, T helper cells, Microenvironment, Infections, Malignant T cell-B cell interaction

## Abstract

Precursor B acute lymphoblastic leukemia (BCP-ALL), the most common childhood malignancy, arises from an expansion of malignant B cell precursors in the bone marrow. Epidemiological studies suggest that infections or immune responses to infections may promote such an expansion and thus BCP-ALL development. Nevertheless, a specific pathogen responsible for this process has not been identified. BCP-ALL cells critically depend on interactions with the bone marrow microenvironment. The bone marrow is also home to memory T helper (Th) cells that have previously expanded during an immune response in the periphery. In secondary lymphoid organs, Th cells can interact with malignant cells of *mature* B cell origin, while such interactions between Th cells and malignant *immature* B cell in the bone marrow have not been described yet. Nevertheless, literature supports a model where Th cells—expanded during an infection in early childhood—migrate to the bone marrow and support BCP-ALL cells as they support normal B cells. Further research is required to mechanistically confirm this model and to elucidate the interaction pathways between leukemia cells and cells of the tumor microenvironment. As benefit, targeting these interactions could be included in current treatment regimens to increase therapeutic efficiency and to reduce relapses.

## Introduction

Precursor B cell acute lymphoblastic leukemia (BCP-ALL) is the most common childhood malignancy and represents the leading cause of cancer-related death in children and young adults [1]. BCP-ALL arises from a monoclonal or oligoclonal expansion of malignant B cell precursors in the bone marrow. The malignant cells are characterized by chromosomal alterations leading to the expression of mutant proteins that confer survival and proliferation advantages. Nevertheless, these genetic lesions are not sufficient for BCP-ALL development. This is suggested by the fact that precursor B cells carrying such characteristic mutations are frequently found in newborns but the prevalence of leukemia is approximately a hundredfold lower [2, 3]. Based on these observations, a two-step model was proposed according to which the leukemia-initiating genetic lesion occurs in utero, followed by an event that promotes expansion of the pre-leukemic clone and eventually leads to the emergence of leukemia. Multiple causes for such a second event have been suggested, and most probably several of them account for the eventual transition to leukemia. Interestingly, epidemiological studies provide evidence that infections or immune responses to infections may represent a major trigger for the leukemia pathogenesis.

In the late 1980s, Kinlen noted a temporal increase in childhood leukemia in several occasions where previously isolated populations mingled [4–8]. At the same time, Mel Greaves postulated a “delayed infection” hypothesis, according to which the development of leukemia is partly caused by an abnormal immune reaction to a common infectious agent [9]. Thereafter, several large studies reported that children who attended a playgroup during their first year of life showed a significant protection from childhood ALL [10, 11]. Thus, similar to the hygiene hypothesis in allergy and asthma, a delayed exposure to common pathogens in developed societies may lead to abnormal or dysregulated immune responses that promote growth of the leukemic clone [12]. Most recently, this model received mechanistic support by an elegant investigation showing that experimental mice predisposed for BCP-ALL development due to a PAX5 mutation only developed BCP-ALL upon transfer from specific pathogen-free (SPF) environment to an environment containing common pathogens [13].

Immune responses to pathogens are typically composed of concerted actions by several types of immune cells. T helper (Th) cells play a central role in orchestrating immune responses by instructing and activating other immune cells. B cells, for example, depend on interaction with Th cells for their survival, proliferation, differentiation to plasma cells, hypermutation, class-switch recombination, adhesion, and migration [14]. While central in a functional immune system, such Th cell-B cell interactions, however, can also contribute to the pathogenesis of lymphoma and leukemia.

Malignantly transformed B cells often retain their capacity to interact with Th cells. As a consequence, malignant B cells seem to profit from Th cell help similar to healthy B cells. Such interactions between Th cells and malignant B cells with untoward effects have been described for several cancers arising from *mature* B cells, the main targets of Th cells. In contrast, the interaction of Th cells with malignant *immature* B cells such as BCP-ALL cells has not been studied extensively. In this article, we review the literature concerning the role of Th cells in mature B cell malignancies and summarize data hinting at a role of Th cells in BCP-ALL, i.e., in *immature* B cells, all in the context of the theory of an infectious etiology of BCP-ALL.

## Review﻿

### Role of the microenvironment in BCP-ALL

The tumor microenvironment plays a key role in supporting survival and expansion of cancer cells [15–17]. In BCP-ALL, a variety of bone marrow stromal cells are believed to support survival and proliferation of BCP-ALL cells [18–21] and to confer drug resistance leading to treatment failure or disease relapse [22, 23]. Mesenchymal stromal cells [24], bone marrow endothelial cells [25], osteoblasts [26], and adipocytes [27] have all been shown to interact with BCP-ALL cells in mechanisms involving both soluble factors like cytokines, chemokines, and growth factors [28–33] as well as cell membrane-bound molecules such as Galectin-3 [34] or VE-cadherin [35]. These crosstalks between leukemic cells and cells of the tumor microenvironment include signaling pathways such as Notch signaling [36] or the wnt pathway [37]. While the microenvironment supports leukemia cells, the leukemia cells, in turn, shape the microenvironment according to their own benefit [38–41]. As a consequence, the bone marrow of leukemia patients exhibits substantial alterations that lead to support of the malignant cells and to impaired hematopoiesis [42].

The bone marrow is also home to mature Th cells [43–45]. These Th cells are derived from a past immune response in the periphery, where they have expanded and subsequently migrated to the bone marrow in order to provide long-term memory allowing for raising a rapid memory response upon re-challenge [46–48]. In addition, these bone marrow Th cells play a crucial role in normal hematopoiesis through the secretion of cytokines and chemokines [49–51].

## Involvement of Th cells in B cell malignancies

Physiological T cell help for B cells takes place in germinal centers in peripheral lymphoid organs, where follicular Th cells interact with mature antigen-stimulated B cells. This interaction involves membrane-bound molecules like CD40 on the B cells and CD40L on the Th cells but also soluble factors like cytokines, chemokines or B cell-activating factor (BAFF) and Fms-related tyrosine kinase 3 (flt3) ligand. Besides providing a suitable environment for the interaction of Th cells and B cells, germinal centers are also the site where malignant transformation of B cells occurs most frequently. This has led to the hypothesis that Th cells may not only support normal germinal center B cells but also germinal center cell-derived malignant B cells. In fact, there is increasing evidence for supportive role of Th cells in mature B cell malignancies. Follicular lymphoma (FL) is a lymphoma of B cells residing in follicles of secondary lymph nodes. FL cells showed an increased survival when stimulated by CD40 crosslinking in vitro [52] as well as upon cognate interaction with CD4^+^ Th cells [53]. Support of FL cells by Th cells was also observed in vivo and seems to be mediated by follicular Th cell-derived CD40L and IL-4 [54]. Hodgkin lymphoma—another B cell malignancy presumably arising from germinal center B cells—is characterized by infiltration of Th cells. Even though the function of these infiltrating Th cells is unclear, the presence of certain Th cells subset has been correlated with reduced overall survival, suggesting that these infiltrating Th cells may support growth of the malignant B cells [55, 56].

Chronic lymphocytic leukemia (CLL) is a malignancy of mature B cells, although the precise cell of origin is unclear [57]. CLL cells proliferate in pseudofollicles in secondary lymphoid organs and in the bone marrow, where they receive support from the microenvironment. Recently, we demonstrated that peripheral blood and lymph nodes of CLL patients contained memory Th cells that were specific for endogenous CLL antigens and were able to interact with CLL cells in an antigen-dependent manner [58, 59]. These Th cells had a Th1-like phenotype and supported autologous CLL cell proliferation in vitro and in murine xenograft experiments. Furthermore, interaction of CLL cells with autologous Th cells led to an upregulation of the risk marker CD38 in an interferon (IFN)-γ-dependent mechanism [60]. Thus, while the support of normal mature B cells is central for the generation of an adaptive immune response, the same interaction between Th cells and malignant B cells seems to promote lymphoma or leukemia derived from malignantly transformed mature B cells.

## A supportive role for Th cells in BCP-ALL?

Unlike the above-mentioned B cell malignancies that originate from mature B cells, BCP-ALL cells derive from precursor B cells, i.e., immature B cells. Surprisingly, little is known about the influence of Th cells on both normal and malignant precursor B cells in the bone marrow. Intriguingly, BCP-ALL cells as well as normal precursor B cells express all molecules required for cognate interaction with Th cells: CD40 [61], major histocompatibility complex (MHC) class II, adhesion and co-stimulatory molecules [62, 63], and receptors for cytokines [64–71] and BAFF [72, 73]. Thus, they seem to have the capacity to present antigen to Th cells and receive support through the classical pathways.

In fact, BCP-ALL cells are receptive for CD40L stimulation, which generally has an activating effect on BCP-ALL cells, inducing proliferation [74] and upregulation of surface molecules like CD70 [75] and the receptor for IL-3 [76], a cytokine with proliferative function in BCP-ALL. In addition, CD40L stimulation was shown to induce secretion of chemoattractants [77] and to upregulate antigen-processing machinery components [78]. This suggests that BCP-ALL cells may attract Th cells and activate them through presentation of antigens, thereby inducing a positive feedback loop.

BCP-ALL cells can also respond to Th cell cytokines. While IL-2 has been found to stimulate proliferation [66], the Th2 cytokines IL-4 and IL-13 instead inhibited growth [74, 79–81], and IL-4 as well as TGF-β induced BCP-ALL cell apoptosis [82–84]. More recently, a proliferative effect of the cytokines IL-17 and IL-21 on BCP-ALL cells has been reported [85]. The observations that cytokines may be involved in the pathogenesis of BCP-ALL are consistent with the highly inflammatory environment in the bone marrow of leukemia patients [40].

Further support for the ability of BCP-ALL cells to react to soluble and membrane-bound Th cell factors comes from a report where stimulation with activated allogenic Th cells induced activation and maturation of BCP-ALL cells [86]. Interestingly, BCP-ALL is associated with certain MHC class II haplotypes. This may be a further hint that antigen presentation to Th cells is involved in BCP-ALL, even though mechanistic evidence is not available yet [87, 88].

## Model for Th cell contribution to BCP-ALL

**Fig. 1:**
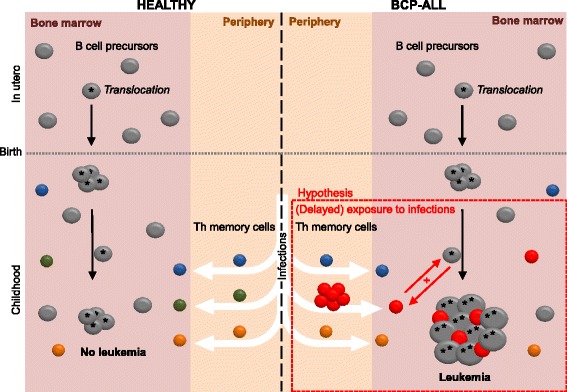
Model integrating the infectious etiology hypothesis with the potential role of Th cells in BCP-ALL pathogenesis. Precursor B cells develop in the bone marrow, where they may undergo chromosomal rearrangements. Cells harboring such translocations that confer survival advantages are often present as expanded clones at birth, but this does not necessarily lead to leukemia development (*left side*). Infections in early childhood induce expansion of Th cells, which home to the bone marrow after the infection has been cleared to take part in normal hematopoiesis and to rise a memory response upon re-challenge with the pathogen (*left side*). Th cells expanded during an aberrant immune response due to delayed pathogen exposure may aberrantly interact with precursor B cells or leukemia cells or both after migration to the bone marrow, supporting their growth and survival, which ultimately leads to leukemia (*right side*)

While epidemiological as well as experimental animal studies suggest that infections contribute to the pathogenesis of BCP-ALL, a specific infectious agent has not been identified. A strong association of BCP-ALL with viral infections such as chicken pox, rubella, measles, and influenza was described in British children [89], but no incorporation of microbial genetic information into host DNA could be detected when 20 BCP-ALL cases where analyzed by representational difference analysis [90]. This makes it unlikely that an infectious agent contributes to BCP-ALL by direct transformation of the precursor B cells. Even though pathogens may stimulate proliferation of precursor B cells or BCP-ALL cells through Toll-like receptors (TLR), this seems rather unlikely, since BCP-ALL cells home to the bone marrow, whereas most pathogens are encountered in the periphery. Instead, Th cells that have expanded during an immune response—possibly due to a delayed first exposure to pathogens—may aberrantly interact with and support the pre-leukemic or leukemic precursor B cells upon (Fig. 1). This interaction may be antigen-independent; alternatively, pathogen-specific Th cells might cross-react with endogenous antigen expressed and presented by the BCP-ALL cells. The nature of such endogenous antigens remains highly speculative and elusive. Attractive candidates are the fusion proteins generated by the characteristic chromosomal translocations, since they are likely to be recognized as foreign as no immune tolerance against these novel proteins has been induced. Indeed, Th cell clones against the fusion proteins TEL/AML1 and BCR/ABL could be generated in vitro [91, 92]. Nevertheless, it remains to be determined whether BCP-ALL patients actually carry such fusion protein-specific Th cells in their bone marrow as expanded clones, and whether these Th cells are able to interact with and support BCP-ALL cells.

In our work on CLL, we found that the CLL-specific Th cells recognized an antigenic peptide within the CLL B cell receptor (BCR) [58]. While not all subsets of BCP-ALL cells express surface pre-BCR, most express components of the pre-BCR intracellularly. Indeed, epitopes within the variable regions of the pre-BCR are apt candidates to engage Th cells, since they are likely to be presented on MHCII, and the Th cells are presumably not tolerant to these peptides. Still, both the antigen-dependence of the interactions between Th cells and BCP-ALL cells as well as the antigenic source remain speculations.

## Implications for therapeutic approaches

Although modern treatment has reached an excellent rate of success in western world, treatment success in high-risk groups such as children with *BCR-ABL* or *MLL-AF4* translocations remains poor. Furthermore, relapse occurs in about 20% of the patients, and the cure rate of a recurrent disease is significantly lower. Survivors often suffer from severe chronic health problems due to the toxic effects current therapy still has [93]. Therefore, there is justified need for biologically targeted therapeutic strategies with less side effects. The tumor microenvironment plays a central role in supporting tumor cell survival, proliferation, and drug resistance. As a consequence, effective leukemia therapies also ought to target the malignant crosstalk between leukemia cells and the supporting cells. Should the leukemia supporting role of Th cells be confirmed, it will be of great importance to elucidate the mechanisms underlying this malignant collaboration, since key molecules of this interaction may be targeted by future therapies. If the interaction of Th cells and BCP-ALL cells is antigen-specific, the BCP-ALL-supporting Th cells are likely to be oligoclonal. Thus, TCR-specific therapeutic antibodies could be generated and used to specifically deplete the leukemia-promoting Th cells, while Th cells with other specificities will remain functional. Although it will not be feasible to apply such a personalized therapy in the initial phase of the treatment, the approach may be combined with the existing drugs in the initial regimen in order to prevent drug resistance and relapse.

## Conclusions

The link between BCP-ALL and infections in early childhood was proposed decades ago, but the pathological mechanisms remain unclear. A direct transformation of B cell precursors by pathogens seems unlikely. Instead, immune cells activated and expanded in response to those pathogens may supportively interact with B cell precursors and thereby promote leukemia. Due to their role in the immune system and their presence in the bone marrow, Th cells are good candidates for such leukemia-supportive immune cells. Indeed, it has been reported that BCP-ALL cells are receptive for soluble and membrane-bound Th cell stimuli. Nevertheless, it is to date unclear if BCP-ALL cells are able to receive help from autologous Th cells, and whether such a supportive interaction actually takes place in BCP-ALL patients’ bone marrow. The tumor microenvironment plays a key role in supporting malignant cells. As a consequence, efficient anti-cancer treatment should include targeting the cells of the microenvironment. Thus, identification and characterization of malignant collaboration between Th cells and BCP-ALL cells or their precursors may provide mechanistic support of the infectious etiology hypothesis and thereby open for novel therapies aiming to target the tumor microenvironment.

## Abbreviations

BAFF: B cell-activating factor
BCP-ALL: Precursor B acute lymphoblastic leukemia
CLL: Chronic lymphocytic leukemia
FL: Follicular lymphoma
IFN: Interferon
MHC: Major histocompatibility complex
Th: T helper
